# How can science be well-ordered in times of crisis? Learning from the SARS-CoV-2 pandemic

**DOI:** 10.1007/s40656-020-00348-5

**Published:** 2020-11-03

**Authors:** Thomas A. C. Reydon

**Affiliations:** Institute of Philosophy & Centre for Ethics and Law in the Life Sciences, Leibniz University Hannover, Im Moore 21, 30167 Hannover, Germany

**Keywords:** Common good, Well-ordered science

## Abstract

The SARS-CoV-2 pandemic constituted a crisis situation in which science was very far from Kitcher’s ideal of well-ordered science. I suggest that this could and should have been different. Kitcher’s ideal should play a role in assessing the allocation of research resources in future crisis situations, as it provides a way to balance highly divergent interests and incorporate the common good into decision-making processes on research.

The SARS-CoV-2 pandemic constituted a crisis situation in which the common good was threatened on a global scale, and in which it was deeply unclear how the needs of all could be met.^1^ It also raised urgent questions regarding the global allocation of resources for research, as a multitude of possible vaccines, cures and public health measures were explored simultaneously and financial support from funding agencies was diverted from various areas of biomedical research (such as translational medicine, basic biomedical research, oncology, etc.) to research on the SARS-CoV-2 virus and the disease it causes, COVID-19. However, with the efforts of the WHO as possibly the only exception, no global debates on these issues were held. Governments and companies each pursued their own research and development programs for vaccines and cures, local governments implemented their own measurements, and different interests were pitted against each other (consider the discussions in the EU, the UK and the USA on keeping mortality rates down vs. reopening economies). There was virtually no worldwide dialogue about the common good in this crisis. But as pandemics are by definition global problems, there *should* have been such a dialogue. We should have done better. How can we do better next time?

I want to suggest that one way of doing better is to explicitly pose the question what the common good in a crisis such as the SARS-CoV-2 pandemic encompasses, and how during such a crisis and its aftermath research can be directed toward accommodating the needs of all. The tool to use to do this, I suggest, is Philip Kitcher’s ideal of *well-ordered science*. Kitcher’s ideal is a response to the question how, given the immense number of topics that could be investigated and the fact that resources for research (funding, humanpower, equipment, time, and so on) are intrinsically limited, research priorities should be set. He developed the ideal in several works (Kitcher, 2001, 2004, 2011; Reiss & Kitcher, 2009; Barker & Kitcher, 2014: Chapter 6) and it has come to play an important role in philosophical debates on responsible research and the governance of science.

Kitcher’s ideal involves the notion of an “ideal deliberation” aimed at promoting the common good. Participants in such a deliberation start out with their own set of (epistemic and non-epistemic) interests and their own list of research priorities, and adjust their priorities as they obtain new information about the current state of scientific knowledge and about the various problems, values and interests of all the various groups in their society. Such a deliberation would result in a ranking of research topics made by a body of decision-makers that has given fair consideration to all viewpoints present in society. Because the ranking results from an unbiased weighing of the various problems, values and interests that are present in society, it can be taken to reflect the common good of this society as a whole. As Kitcher (2004: 333) writes: “Science is well-ordered when the inquiries it pursues are those that accord with the agenda that would have been set by a group of discussants fully informed of the scientific opportunities, fully informed of one another’s needs, and dedicated to doing the best they can to accommodate the needs of all.”

What, then, was the common good in the SARS-CoV-2 pandemic? While the problem in a pandemic primarily consists of an infectious disease, it does not only directly affect the lives of people who were or will be infected, but also of almost everyone else on the planet due to a large diversity of secondary effects. These include mental health issues or loss of income due to lockdowns; longer-term financial problems and losses of opportunities due to local and global economic recessions; infractions of human rights due to increased monitoring of citizens, travel restrictions, shelter-in-place orders, etc.; increases in domestic violence; educational backlogs for students and additional burdens for parents due to the closure of educational institutions and the need for homeschooling; and many more. Thus, even though the SARS-CoV-2 pandemic was caused by a biomedical phenomenon–an infectious disease–it in fact was a highly multifaceted problem. Accordingly, resolving it and, equally importantly, increasing preparedness for a likely next time do not only require biomedical research on vaccines, cures and pathways of infection, but also research on a multitude of other issues: on how different socioeconomic groups were affected differently by the pandemic, on local and global economic effects, on the legitimacy of restricting individual rights by enforced lockdowns to promote public health, on possible effects of homeschooling and online teaching on students’ educational progress, on effective communication about science and policy measures, and much more.

The needs of all and the research efforts that could be undertaken to meet them, thus, are a very diverse lot. While (almost) all members of the human population have strong interests in ending the pandemic and mitigating its downstream consequences, different people were, are or may in (even the more distant) future be affected differently and thus have interests in different aspects of the problem. Indeed, for different people the SARS-CoV-2 pandemic will constitute a *different kind* of problem or, as individuals may be confronted with multiple effects of the pandemic, combination of problems: for infected people it might be foremost a medical problem (depending on whether they develop any symptoms), for parents it might be foremost a problem of how to provide their children with a good education, for someone who lost their job because of a lockdown it will primarily an economic problem, and so on. Because there are so many different interest at stake and so many different possible research topics are related to these interests in complicated ways, the task of directing the allocation of research resources toward the needs of all is daunting.

Kitcher’s ideal can be a useful tool in this context, precisely *because* it is an ideal. In practice, any decision-making processes will fall well short of an ideal deliberation because the ideal presupposes all participants in the debate to be ideal decision makers: fully open to change their views, fully empathic to the plights of others, fully informed about the nature of the problem, the various ways in which others are affected, and the state of the art in science and technology, etc. Real people of course aren’t such ideal decision makers and even if they were, there are too many divergent interests to reach a fair weighing. But this does not render the ideal of well-ordered science useless: it can still serve as a beacon on which *individual* scientists, policy makers, interest groups, and other stakeholders can orient themselves (without actually conducting any debate) by asking themselves what, given their current state of information, the outcome of an ideal deliberation *would* or *could* have been, and prioritize research efforts accordingly. Note that this suggestion does not involve actual debates within societies, in which only the interests that are present in the society in which the debate occurs are voiced and heard. Rather, on this suggestion individual people and institutions consider which interests could exist, how they should be weighed and how research could be best attuned to these weighed interests. This allows a wide variety of interests, approaches and values to enter into the considerations, not limited to those in a particular society or in the Global North (to which discussions on scientific research are often limited) but equally including the Global South. Scientific research should serve all of humanity equally and be conducted by researchers from all regions of the planet, and using Kitcher’s ideal as a tool for individual considerations and individual decision making rather than debates involving an entire society is a way to achieve more inclusivity in the allocation of resources for research.

Of course, this will not yield an allocation of research resources that really accommodates the needs of all. But it will at least prompt researchers, institutions and other stakeholders to ask what *their* best contribution to addressing the needs of all could be, thus bringing considerations about the common good into individual decisions about research. While this cannot and should not replace an ongoing global dialogue about how research can serve the common good in times of crisis, making Kitcher’s ideal more concrete and familiarizing researchers, policy makers and other stakeholders with it (which both are tasks for philosophers of science) will be a first step in the right direction. A general lesson that can be drawn from the SARS-CoV-2 pandemic, then, pertains to how work in the philosophy of science can help us cope with–and be better prepared for–crisis situations.

## Footnotes

1 This note belongs to the Topical Collection “Seeing Clearly Through COVID-19: Current and future questions for the history and philosophy of the life sciences”, edited by G. Boniolo and L. Onaga.
